# Efficacy and Safety of Thirty-Day Dual-Antiplatelet Therapy Following Complex Percutaneous Coronary Intervention: A Systematic Review and Meta-Analysis

**DOI:** 10.3390/jcdd11020043

**Published:** 2024-01-29

**Authors:** Anastasios Apostolos, David-Dimitris Chlorogiannis, Grigorios Chrysostomidis, Maria Bozika, Filippos Timpilis, Angelos Kramvis, Grigoris V. Karamasis, Georgios Leventopoulos, Periklis Davlouros, Grigorios Tsigkas

**Affiliations:** 1 First Department of Cardiology, Hippocration General Hospital, National and Kapodistrian University of Athens, 157 72 Athens, Greece; anastasisapostolos@gmail.com; 2 Department of Radiology, Brigham and Women’s Hospital, Boston, MA 02115, USA; 3 Department of Adult Cardiac Surgery, Onassis Cardiac Surgery Center, 176 74 Athens, Greece; gregory.chrisostomidis@gmail.com; 4 Department of Cardiology, University Hospital of Patras, 265 04 Patras, Greece; mariabozika29@gmail.com (M.B.); philtimpi99@gmail.com (F.T.); angeloskramvis@gmail.com (A.K.); levent2669@gmail.com (G.L.); pdav@upatras.gr (P.D.); 5 Second Cardiology Department, Attikon University Hospital, National and Kapodistrian University of Athens Medical School, Rimini 1, Chaidari, 124 62 Athens, Greece; grigoris.karamasis@gmail.com

**Keywords:** dual-antiplatelet therapy, complex percutaneous coronary interventions, bifurcations, chronic total occlusion, PCI

## Abstract

The optimal duration of DAPT after complex PCI remains under investigation. The purpose of this systematic review and meta-analysis was to explore the safety and efficacy of a one-month therapy period versus a longer duration of DAPT after complex PCI. We systematically screened three major databases, searching for randomized controlled trials or sub-analyses of them, which compared shortened DAPT (S-DAPT), namely, one month, and longer DAPT (L-DAPT), namely, more than three months. The primary endpoint was any Net Adverse Clinical Event (NACE), and the secondary was any MACE (Major Adverse Cardiac Event), its components (mortality, myocardial infarction, stroke, and stent thrombosis), and major bleeding events. Three studies were included in the analysis, with a total of 6275 patients. Shortening DAPT to 30 days after complex PCI did not increase the risk of NACEs (OR: 0.77, 95% CI: 0.52–1.14), MACEs, mortality, myocardial infractions, stroke, or stent thrombosis. Pooled major bleeding incidence was reduced, but this finding was not statistically significant. This systematic review and meta-analysis showed that one-month DAPT did not differ compared to a longer duration of DAPT after complex PCI in terms of safety and efficacy endpoints. Further studies are still required to confirm these findings.

## 1. Introduction

Dual-antiplatelet therapy (DAPT) after percutaneous coronary intervention (PCI) consists of a combination of acetylsalicylic acid and a P2Y12 inhibitor and is crucial for the prevention of coronary thrombotic events [1]. Using DAPT for an extended period and/or using a potent P2Y12 inhibitor, such as ticagrelor or prasugrel, may decrease the residual ischemic risk, but it may also lead to an increase in bleeding events [2,3,4]. The risk of ischemic events following percutaneous coronary intervention (PCI) is directly proportional to the complexity of the procedure, owing to both anatomical and technical factors [5,6,7]. Moreover, patient characteristics, like diabetes mellitus, active cancer, or tobacco smoking, could lead to an increased risk for ischemic events [8,9,10]. However, an extended duration of dual-antiplatelet therapy (DAPT) has been shown to increase the risk of major bleeding, which is associated with elevated rates of morbidity and mortality [11,12,13].

Consequently, determining the ideal duration and combination of DAPT for patients after complex PCI is a matter of ongoing controversy in the interventional cardiology community. The formulation of a universally accepted definition of complex PCI has not been achieved yet; nonetheless, the most prevalent criteria for categorizing PCI as “complex” include previous stent thrombosis, interventions within the last patent major epicardial artery, intervention in multivessel coronary disease, deployment of at least three stents, treatment of at least three distinct lesions, bifurcation lesions stenting with at least two stents, the implantation of stents with a cumulative length exceeding 60 mm, calcified lesions requiring modification, left main angioplasty, PCI in post-CABG patients, and revascularization of chronic total occlusion (CTO) [1].

Current guidelines published by the European Society of Cardiology (ESC) suggest 6 and 12 months of DAPT in stable ischemic heart disease and acute coronary syndromes, respectively, with the option of extending the duration in high-ischemic-risk patients and shortening it in high-bleeding-risk patients by up to 1 month [14,15,16]. Nonetheless, these specific time points can be tailored to each patient’s unique characteristics, weighing factors related to their ischemic and bleeding risk. Currently, there is growing evidence from randomized controlled trials (RCTs) and meta-analyses supporting the safety and effectiveness of earlier stoppage of DAPT, exploring a duration of three or even just one month of DAPT [4,17,18]. However, there is a lack of data as to whether these promising preliminary findings can be translated to high-ischemic-risk patients, such as those with complex PCI. The purpose of this systematic review and meta-analysis was to explore the efficacy and safety of 30-day, shortened DAPT (S-DAPT) in patients undergoing complex PCI and to compare it with DAPT lasting for more than three months (L-DAPT).

## 2. Materials and Methods

This systematic review and meta-analysis was conducted in compliance with the updated Preferred Reporting Items for Systematic Reviews and Meta-Analyses (PRISMA) 2020 statement [19]. The protocol of this study was pre-registered in the Open Science Framework (URL: osf.io/9swby, accessed on 22 January 2024). The quantitative and qualitative analysis was a study-level meta-analysis, and thus institutional board review approval was not required, as this type of analysis involves the synthesis of previously published data from the existing literature and does not involve direct interaction with participants.

### 2.1. Eligibility Criteria and Endpoints

Studies were considered eligible for the specific systematic review and meta-analysis if they met all the following inclusion criteria: (i) randomized controlled studies (RCTs) including human subjects or sub-studies and post hoc analyses of them; (ii) DAPT duration ≤ 30 days in the experimental arm; (iii) DAPT duration > 31 days in the control arm; (iv) available data for patients with previous complex PCI; and (v) bleeding characterization in accordance with Bleeding Academic Research Consortium (BARC) criteria. The definition of complex PCI is not universal, so it was used as per trial definition, as reported in Table 1. The PICO approach is presented in Supplementary Table S1.

The primary endpoint of this study was to assess the incidence of net adverse clinical events (NACE), which is a composite outcome that includes both ischemic and bleeding events. Major Adverse Cardiovascular Events (MACE) is another composite endpoint that includes death, non-fatal myocardial infarction, non-fatal stroke, and stent thrombosis. All the components of MACE were also used as secondary endpoints. Another secondary endpoint was major bleeding, classified as a class 3–5 bleeding event according to Bleeding Academic Research Consortium (BARC) criteria.

Taking into consideration the variability in outcome definitions across the included trials, an overview of these definitions is shown in Supplementary Table S2.

### 2.2. Information Sources

We combed the current literature by conducting a comprehensive, bibliographic database screening of the three main databases: Cochrane Central Register of Controlled Trials (CENTRAL), Medline, and Scopus. All the searches were performed on 25 September 2022 and updated on 10 January 2023. Moreover, we explored the reference list of each included study to recognize any other eligible report.

### 2.3. Search Strategy

The search included these terms: “dual antiplatelet treatment”, “DAPT”, “percutaneous coronary intervention”, “PCI”, “stent”, “complex”, “bifurcation”, “CTO”, and “chronic total occlusion”. The search approach was modified for each database, as it is presented in Supplementary Table S3. No language restrictions were imposed on our protocol.

### 2.4. Selection Process

All records retrieved from the search of libraries were imported into Rayyan, and then duplicates were manually removed [23]. Titles, abstracts, and keywords were screened by two independent referees, and irrelevant reports were excluded. The full text was assessed by two independent reviewers. Each disagreement between them was solved via discussion with the other authors.

### 2.5. Data-Collection Process

To extract study outcomes and patient characteristics, a data extraction form was created and evaluated for appropriateness by all authors using two randomly selected studies. Then, three authors independently obtained data from each report, and a third author validated the data, handled any issues, and entered them into Review Manager 5.4 (Review Manager 2014).

### 2.6. Data Items

The following data were extracted: (A) the report: authors, publication year; (B) the study: population size, inclusion and exclusion criteria, complex PCI definition, endpoints definition; (C) the participants: demographic characteristics, comorbidities, pharmacotherapy; (D) the procedure: peri-procedural characteristics, stent type, indication for intervention, type of lesions; (E) type and duration of DAPT; and (F) outcomes during twelve-month follow-up.

### 2.7. Risk of Bias Assessment

The risk of bias was evaluated with the Cochrane “Risk of bias” tool for randomized trials (RoB 2.0), with two investigators independently applying the tool in each study [24]. In cases of disagreements in the risk of bias assessments, the two authors discussed and reached a consensus, with a third participant serving as an arbiter if required. To investigate potential publication bias, funnel plots were created, plotting the sample size against the odds ratio (OR) for each endpoint.

### 2.8. Statistical Analysis

Every analysis was performed at the study level. The effect of different DAPT regimens was evaluated by estimating OR with 95% confidence intervals (CIs). If a study did not present sufficient data for a specific outcome, it was excluded from the analysis for that specific outcome. Every analysis was performed using an intention-to-treat approach. The pooled OR was calculated by performing the random-effect model (Mantel–Haenzel) [25]. The in-between study heterogeneity was evaluated by applying the statistical inconsistency test (I^2^ = 100% × (Q − df)/Q, where Q = χ^2^ (Cochran’s heterogeneity statistic) and dF = its degrees of freedom), where I^2^ ≤ 25% signifies low, I^2^ ≤ 50% is moderate, and I^2^ > 50% is high heterogeneity [26]. *p*-values < 0.05 were significant. Sensitivity analysis was conducted by removing one study each time and repeating the statistical analysis.

## 3. Results

### 3.1. Search Results

After conducting a systematic search of the three databases, 1651 unique records were identified. Following the removal of duplicates, 1198 records underwent title, keywords, and abstract review. Subsequently, 21 reports underwent full-text screening. Through consideration of the reference lists of these studies, a total of three studies were considered eligible and included in this systematic review and meta-analysis [20,21,22]. Our systematic search of the literature is depicted in the PRISMA flowchart (Supplementary Figure S1).

### 3.2. Studies’ Characteristics

The characteristics of the included studies are shown in Table 1. Each study’s inclusion and exclusion criteria, as well as endpoints, primary or secondary, are presented in Supplementary Table S4. The included reports are sub-studies or post hoc analyses of RCTs. A total of 6275 patients were analyzed; 3116 were treated with S-DAPT, and 3159 were treated with L-DAPT. In MASTER DAPT, the duration of DAPT in the L-DAPT arm was more than 3 and at most 12 months; in GLOBAL LEADERS and STOPDAPT-2, it was 12 months. Both patients with chronic and acute coronary syndromes were included in this analysis. Three-vessel PCI, ≥3 lesions treated, PCI with at least 3 stents, total stent length > 60 mm, and bifurcation PCI ≥ 2 stents were mutual in their complex PCI definition in all three included studies. CTO cases were analyzed in STOPDAPT-2 and MASTER DAPT but not in GLOBAL LEADERS. All endpoints were evaluated during 1-year follow-up.

### 3.3. Patient Characteristics

The baseline characteristics are shown in Table 2. Patients included in MASTER DAPT were older, with a mean age over 75 years; meanwhile, the mean age of patients included in the other two trials was less than 70 years. Women were under-represented in all trials, comprising less than 30% of the total patients. The mean body mass index of the three studies varied between 24.3 and 28.1 kg/m^2^; in STOPDAPT-2, the mean body mass index was normal, and in the other two trials, weight status was overweight. Hypertension was the most frequent comorbidity in patients included in GLOBAL LEADERS and MASTER DAPT; in STOPDAPT-2, most patients suffered from dyslipidemia, while about a quarter of analyzed patients had a previous PCI.

Acute coronary syndrome was not the main indication for PCI in any of the trials, though a significant proportion of patients was catheterized for this indication (30.3–49.1%), mainly through the transradial approach. Data about the utilization of newer physiology (fractional flow reserve) and imaging techniques (intravascular ultrasound and optical coherence tomography) were limited. Nevertheless, according to these data, their usage was limited to less than 20% of the total complex PCI procedures.

### 3.4. Primary Endpoint: Net Adverse Cardiac Events

**Figure 1:**
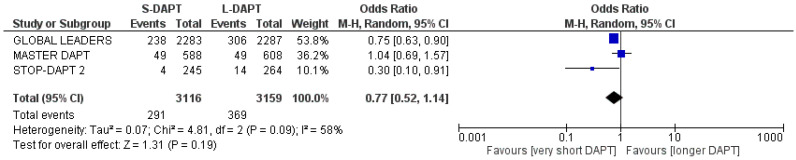
Forest plot demonstrating the effect of very short-term versus more than 3 months dual antiplatelet treatment on net adverse clinical events, with odds ratio and 95% confidence intervals. Each square represents a study; the size of square is representative of the weight of each study. The diamond shows the result when all the particular studies are combined together and averaged. CI, confidence interval; DAPT, dual antiplatelet therapy; M-H, Mantel–Haenszel.

The primary composite = endpoint occurred in 291 patients in the S-DAPT group and in 369 in the L-DAPT. S-DAPT reduced the incidence of NACE, though this result was not statistically significant (OR: 0.77, 95% CI: 0.52–1.14). Heterogeneity was considered as high (I^2^ = 58%) (Figure 1).

### 3.5. Secondary Endpoints

#### 3.5.1. MACE

**Figure 2:**
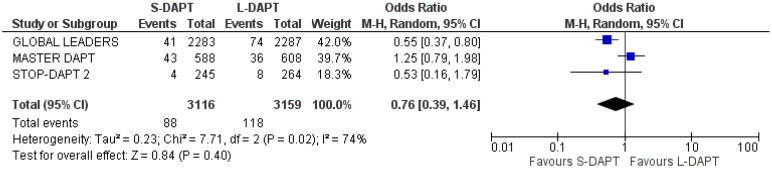
Forest plot demonstrating the effect of very short-term versus more than 3-month dual antiplatelet treatment on major adverse clinical events, with odds ratio and 95% confidence intervals. CI, confidence interval; DAPT, dual antiplatelet therapy; M-H, Mantelel Haenszel; MACE, major adverse cardiovascular events.

All the analyzed studies (n = 3, with a total of 6275 patients) presented data regarding MACE. A total of 88 patients presented with MACE in the S-DAPT group and 118 in the L-DAPT group. The pooled analysis did not show a significant difference between the two groups (OR: 0.76, 95% CI: 0.39–1.46). High heterogeneity (I^2^ = 74%) was observed (Figure 2).

#### 3.5.2. All-Cause Mortality

**Figure 3:**
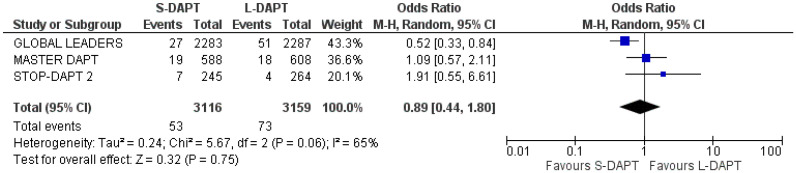
Forest plot demonstrating the effect of very short-term versus more than 3-month dual antiplatelet treatment on all-cause mortality, with odds ratio and 95% confidence intervals. CI, confidence interval; DAPT, dual antiplatelet therapy; M-H, Mantelel Haenszel.

All the analyzed studies (n = 3 with a total of 6275 patients) presented data regarding all-cause mortality. 53 patients presented with MACE in S-DAPT group and 73 in L-DAPT. All-cause mortality did not significantly differ between the two groups (OR: 0.89, 95% CI: 0.44–1.80) (Figure 3). Heterogeneity was considered high (I^2^ = 65%).

#### 3.5.3. Myocardial Infarction

**Figure 4:**
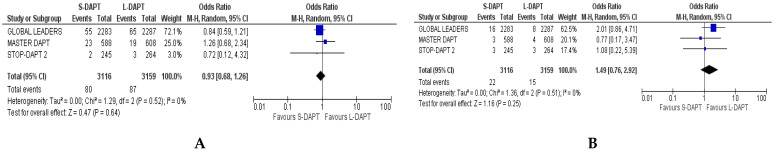
Forest plot demonstrating the effect of very short-term versus more than 3-month dual antiplatelet treatment on myocardial infarction (**A**) and stroke (**B**), with odds ratio and 95% confidence intervals. CI, confidence interval; DAPT, dual antiplatelet therapy; M-H, Mantelel Haenszel.

All included studies presented data about myocardial infarction. We found 80 patients in S-DAPT and 87 patients in L-DAPT who were suffered from MI during 12-month follow-up (OR: 0.93, 95% CI: 0.68–1.26) (Figure 4A). No heterogeneity was calculated regarding myocardial infarction among the studies (I^2^ = 0%).

#### 3.5.4. Stroke

Information about stroke was available for the total population. Non-fatal stroke incidence in the total sample was 0.006%. No significant difference between the two arms was observed (OR: 1.49, 95% CI: 0.76–2.92) (Figure 4B). No heterogeneity was calculated among the analyzed studies (I^2^ = 0%).

#### 3.5.5. Stent Thrombosis

**Figure 5:**
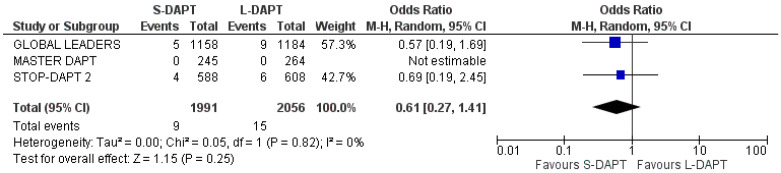
Forest plot demonstrating the effect of very short-term versus more than 3-month dual antiplatelet treatment on stent thrombosis, with odds ratio and 95% confidence intervals. CI, confidence interval; DAPT, dual antiplatelet therapy; M-H, Mantelel–Haenszel.

Stent thrombosis information (definite or probable) was available from all studies. The incidence was similar between the two groups (OR: 1.05, 95% CI: 0.62–1.77) (Figure 5). No heterogeneity was calculated among the analyzed studies (I^2^ = 0%).

#### 3.5.6. Major Bleedings

**Figure 6:**
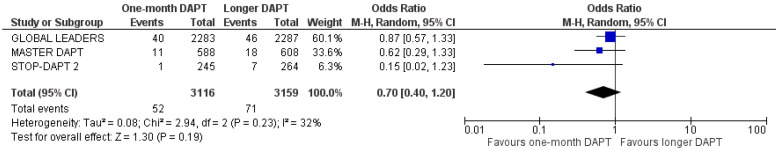
Forest plot demonstrating the effect of very short-term versus more than 3-month dual antiplatelet treatment on major bleedings, with odds ratio and 95% confidence intervals. CI, confidence interval; DAPT, dual antiplatelet therapy; M-H, Mantelel–Haenszel.

All studies provided data about major bleedings, namely, BARC 3-5, during twelve-month follow-up. Among the patients who received S-DAPT, 52 experienced bleeding, compared to 71 individuals in L-DAPT. Despite the numerical decrease in hemorrhagic events in patients randomized to S-DAPT, we did not reveal any significant difference between the two arms (OR: 0.70, 95% CI: 0.40–1.20) (Figure 6). Moderate heterogeneity was observed in this analysis (I^2^ = 32%).

### 3.6. Risk of Bias Assessment

A risk of bias summary and graph following the RoB 2.0 tool is available in Supplementary Figure S2. All the studies were in the lowest risk of bias classifications. Moreover, publication bias was assessed visually with funnel plots. Publication bias was considered low, as a symmetric distribution of the mean effect size for every endpoint was observed (Supplementary Figure S3).

## 4. Discussion

To the best of our knowledge, this is the first systematic review and meta-analysis investigating the effectiveness and safety of one-month DAPT in complex PCI. The present study showed that S-DAPT did not significantly differ in composite endpoints (NACE), ischemic MACE (mortality, myocardial infarction, stroke, and stent thrombosis), or hemorrhagic endpoints (major bleedings classified as BARC 3-5), compared with L-DAPT.

Complex PCI has been established as an independent risk factor for the recurrence of ischemic events, further augmenting the risk posed by known clinical parameters like diabetes mellitus, hyperlipidemia, and tobacco smoking [1,27,28]. Taking this into consideration, Gennaro Giustino et al. compared patients treated with 3–6 month DAPT versus those with >12 months by merging patient-level data derived from six RCTs. A total of 1680 patients having undergone complex PCI were treated with either longer (N = 854) or standard (N = 826) duration of DAPT. Prolonged DAPT for over 12 months significantly reduced the MACE (4.1 vs. 6.8%, adjusted hazard ratio: 0.56, 95% CI: 0.35–0.89), without having an important impact on myocardial infarctions (2.9 vs. 4.8%, adjusted hazard ratio: 0.60, 95% CI: 0.35–1.06), all-cause mortality (3.2 vs. 3.3%, adjusted hazard ratio: 1.11, 95% CI: 0.60–2.04), and major bleedings (1.3 vs. 0.7%, adjusted hazard ratio: 1.81, 95% CI: 0.67–4.91); thus, prolonged DAPT was associated with reduced ischemic events without exposing patients to increased bleeding risk. Moreover, the benefit of prolonged DAPT was greater in interventions with increased complexity [6].

The shift from extended DAPT to abbreviated DAPT has been the subject of ongoing investigation. In this domain, the preliminary results observed in earlier and more recent trials that reduction in recurrent ischemic events observed between earlier and more recent trials can be attributed to several factors. Foremost among these is the critical role played by the choice of antiplatelet agents following the abbreviation of DAPT, affecting the delicate equilibrium between ischemic events and bleeding complications. Two of the studies that were included in this study exclusively used P2Y12 inhibitors after DAPT discontinuation (GLOBAL LEADERS utilized ticagrelor and STOPDAPT-2 clopidogrel). Nevertheless, a small proportion of the MASTER DAPT trial received acetylsalicylic acid. Conversely, in the meta-analysis by Giustino et al., the included trials that showed benefit in the prolongation of DAPT after complex PCI had only acetylsalicylic acid in the interventional arm [6]. Nowadays, clopidogrel is widely used after PCI to offer a better balance of both ischemic and bleeding events than the one provided by acetylsalicylic acid. In this context, the HOST-EXAM trial demonstrated a favorable net clinical benefit of sustained clopidogrel monotherapy when compared to acetylsalicylic acid alone in patients undergoing coronary PCI with drug-eluting stents. A sub-analysis of the HOST-EXAM trial for complex PCI did not reveal any significant difference but only a numerical trend in favor of the clopidogrel group. However, it was not designed for evaluating this sample, and concerns about sample size bias were raised, as the sample size calculation was not performed in this setting [29]. Similarly, the HOST-EXAM Extended study, which was designed to perform a post-trial extended follow-up after the first two years of the same patient cohort as the HOST-EXAM trial, showed a clear benefit of clopidogrel administration as it was associated with a 41% reduced hazard ratio in both ischemic and bleeding events [30]. The previously described reduction in ischemic events could be attributed to the shift toward more potent antiplatelet agents.

Furthermore, the temporal aspect of conducting RCTs may also have had an influential impact on the results. The present meta-analysis encompassed studies that initiated patient recruitment after 2013, in contrast to Giustino et al., who analyzed earlier clinical trials that began patient recruitment as far back as 2009. Over the years, there have been substantial advancements in cardiovascular technology. The three studies included in this systematic review and meta-analysis have utilized only newer generations of drug-eluting stents, as described in Table 1.

Additionally, the integration of intravascular imaging modalities, intravascular ultrasound (IVUS) or optical coherence tomography (OCT), has gained substantial ground in the management of complex atherosclerotic lesions due to their well-documented correlation with improved clinical results [31]. A plethora of studies have substantiated the clinical significance of IVUS-guided PCI in enhancing short-, mid-, and long-term clinical and angiographic outcomes [32,33,34]. Against this background, Hannan et al. observed a progressive escalation in the utilization of IVUS-guided PCI among patients presenting complex vascular lesions, surging from 13.4% in 2014 to 16.5% in 2018 within New York-based healthcare facilities [35]. These observations align with those made by Mentias et al., who reported a discernible uptick in the utilization of IVUS performance in the context of PCI, which rose from 3.0% in 2009 to 6.9% in 2016 within the United States. Similarly, optical coherence tomography (OCT) became commercially available for the assessment of coronary artery plaques around 2009, resulting in its gradual and sustained clinical adoption over the subsequent years [36]. OCT affords high-fidelity visualization of the vascular wall, lumen, constituent elements of plaques, potential dissections, and stent structures, enabling meticulous measurements and optimization in the intricate realm of percutaneous coronary intervention. Consequently, the implementation of OCT-guided complex PCI may yield discernible clinical advantages [34,37]. Indeed, OCT usage has gained important ground in clinical practice, as its application has sextupled between 2010 and 2019 [38]. The utilization of IVUS and OCT in complex PCI in each trial has been presented in Table 2. Furthermore, the methodologies employed in the field of interventional cardiology have undergone remarkable enhancements, leading to enhanced stent placement, reduced incidence of stent thrombosis, and decreased occurrences of ischemic complications. Novel techniques geared toward the management of coronary bifurcations have been devised, subjected to rigorous evaluation through randomized controlled trials, and executed during the past decade [39,40]. Consequently, there has been a consistent augmentation in the rates of successful chronic total occlusion revascularization. As supported by Zein et al., the comprehensive procedural success rate exhibited an enhancement from 45% in 2010 to 55% in 2017 [41]. More recent data from the PROGRESS-CTO International Registry showed an increased success rate over time, from 81.6% in 2016 to 88.1% in 2021 (*p* < 0.0001) [42]. Fellowships and educational courses, like Complex, Higher-Risk, and Indicated Percutaneous Coronary Intervention (CHIP), could play a pivotal role in enhancing clinical knowledge and providing practical training, resulting in improved procedural outcomes [43]. All these may increase our understanding as to why DAPT shortening could drive the reduction in post-angioplasty ischemic adverse events.

Moreover, it is theorized that a shorter duration of DAPT could drive fewer bleeding events. In the present analysis, a numerical decrease in hemorrhagic events was observed, but it did not reach statistical significance (OR: 0.70, 95% CI: 0.40–1.20). Paradoxically, previous meta-analyses included both 30 days and 3 months in S-DAPT have shown bleeding risk reduction [43,44,45,46]. More specifically, this meta-analysis, which included five studies and a total of 9115 patients, showed a 43% odds reduction without having an impact on ischemic events or mortality [47]. Nicolas et al. conducted a meta-analysis, showing that either one- or three-month DAPT significantly reduced bleeding events in both patients undergoing complex (HR: 0.66, 95% CI: 0.44–0.98) and non-complex PCI (HR: 0.60, 95% CI: 0.45–0.79). Against the same background, an individual patient-data meta-analysis by Gragnano et al. showed that less than three months of DAPT tailored with P2Y12 monotherapy reduced BARC 3 or 5 bleeding events in patients undergoing complex PCI (HR: 0.51; 95% CI: 0.31–0.84) [48,49,50,51,52]. The reasons why 30 days did not reduce bleeding events, while 1- or 3-month DAPT did, remains a subject of investigation, which could partially be explained by decreased statistical power due to the smaller sample size or to the more potent agent chosen as monotherapy after one-month DAPT. Of note, in the GLOBAL LEADERS trial, which had the highest weight for major bleeding in the pooled analysis, 100% of patients received ticagrelor.

This systematic review and meta-analysis showed that S-DAPT displays equivalent bleeding, mortality, and ischemic events in a mixed population undergoing complex PCI; a clearer benefit could be expected in high-bleeding risk patients. The clinical advantages of DAPT duration shortening have been substantiated in meta-analyses encompassing both individuals at high and low risk of bleeding events. As previously shown, one-month DAPT reduces major bleedings by 22% (OR: 0.78, 95% Cl: 0.65–0.94) and one or three months of DAPT by 29% (OR 0.71, 95% Cl 0.61–0.82), without having a negative impact on mortality, composite outcomes, or ischemic events, respectively [8,20,53,54]. Nonetheless, the benefits are more pronounced in high-risk bleeding patients, as evidenced by the results of two recent meta-analyses. Costa et al. demonstrated that a DAPT regimen lasting one to three months is linked to decreased bleeding incidents and reduced cardiovascular mortality [55]. Garg et al. analyzed six RCTs and three propensity-matched studies including 16,848 patients. It was found that major bleeding incidence was significantly lower with S-DAPT (OR: 0.68, 95% CI 0.51–0.89), whereas MI did not differ significantly between the two groups (OR: 1.16, 95% CI: 0.94–1.44) [4].

Taking into consideration that a significant part of analyzed patients are classified as high bleeding risk, one-month DAPT and maintenance with P2Y12 inhibitor after complex PCI could be applied with greater confidence under special circumstances, for example, in the event of unplanned DAPT cessation for an urgent surgery.

This study is not without limitations. First of all, this is a study-level meta-analysis; thus, it was not feasible to perform a patient-level analysis. Additionally, it includes patients presenting with either acute or chronic coronary syndromes who have different ischemic and hemorrhagic risks. Furthermore, the term “complex PCI” encompasses a spectrum of PCI settings, making it impractical to conduct sub-analyses for each definition. Nevertheless, all studies demonstrated low-to-moderate heterogeneity, suggesting that the diverse clinical presentations did not significantly impact the findings. Additionally, different P2Y12 inhibitors were used in each study (Table 1). Lastly, it exclusively comprised subgroup and post hoc analyses derived from randomized controlled trials (RCTs), thereby making the possibility of subgroup-related phenomena difficult to exclude.

## 5. Conclusions

This systematic review and meta-analysis showed that one-month DAPT did not significantly differ compared to a longer duration of DAPT after complex PCI in terms of safety and efficacy. Further trials focusing on complex PCI are required to confirm these findings.

## Figures and Tables

**Table 1:** Study characteristics.

No.	Trial	Year	Duration	Time of Randomization	S-DAPT		L-DAPT		Stent Used	Definition of Complex PCI
					Regimen	Continuation with	Regimen	Duration (Months)		
1	GLOBAL LEADERS [20]	2019	2013–2015	At index PCI	ASA 75–100 mg qd + Ticagrelor 90 bid	Ticagrelor 90 mg bid	ASA 75–100 mg qd + Ticagrelor 90 bid/clopidogrel 75 mg qd	12	Biolimus A9-eluting stent	At least one of the following: Three vessels treated; ≥3 lesions treated; ≥3 stents implanted; TSL > 60 mm; Bifurcation with 2 stents implanted
2	STOPDAPT-2 [21]	2021	2015–2017	At 1 month	ASA 81–100 mg qd + clopidogrel 75 mg qd or prasugrel 3.75 qd	Clopidogrel 75 mg qd	ASA 81–100 mg qd + clopidogrel 75 mg qd	12	cobalt-chromium everolimus-eluting stent	Three vessels treated; ≥3 lesions treated; ≥3 stents implanted; TSL > 60 mm; Bifurcation with 2 stents implanted; CTO
3	MASTER-DAPT [22]	2022	2017–2019	At 1 month	ASA + clopidogrel or Ticagrelor or prasugrel	NA	ASA + clopidogrel or Ticagrelor or prasugrel	3–12	Bioresorbable Polymer Coated Stent	At least one of the following: Three vessels treated; ≥3 lesions treated; ≥3 stents implanted; TSL > 60 mm; Bifurcation with 2 stents implanted; CTO

ACS; Acute Coronary Syndrome; ASA; Acetysalycic Acid, CCS; Chronic Coronary Syndrome, CTO; Chronic Total Occlusion, L-DAPT; longer dual antiplatelet therapy, S-DAPT; Short Dual Antiplatelet Treatment, PCI; Percutaneous Coronary Intervention, TSL; total stent length.

**Table 2:** Patients’ characteristics.

	GLOBAL LEADERS [20]		STOPDAPT-2 [21]		MASTER DAPT [22]	
	S-DAPT	L-DAPT	S-DAPT	L-DAPT	S-DAPT	L-DAPT
Patients (n)	2283	2287	245	264	588	608
Age (years)	65.3	65.2	69.2	69.8	76.5	76.8
Female (%)	21.8	20.9	18.8	20.8	28.7	29.6
BMI (kg/m^2^)	28.0	28.1	24.5	24.3	27.6	27.6
Smoking (%)	26.9	26.5	22.9	20.8	8.0	10.8
HTN (%)	74.5	73.0	75.9	76.5	80.4	75.7
DM (%)	27.5	25.1	45.3	50.0	34.4	32.3
Dyslipidemia (%)	69.8	71.2	78	80.3	71.4	65.7
PAD (%)	6.4	7.3	6.9	8.0	12.8	9.8
CKD (%)	14.1	14	37.1	36.7	22.3	16.8
Previous PCI (%)	29.3	29.4	23.3	23.1	27	25.5
ACS (%)	48.6	48.6	34.3	30.3	49.1	47.3
STEMI (%)	13.9	13.9	18.0	16.3	10.0	10.7
NSTEMI (%)	23.3	22.9	4.9	5.7	29.3	26.6
UA (%)	11.3	11.8	9.8	11.4	8.8	10.0
No. of stents (n)	NA	NA	2.74	2.69	3.2	3.1
Total length of stents (mm)	NA	NA	74.4	72.2	76.0	74.1
Transradial approach	75.6	73.9	82.4	81.8	82.0	83.1
FFR	NA	NA	16.7	14.8	NA	NA
OCT	NA	NA	10.6	11.0	3.9	4.9
IVUS	NA	NA	93.5	95.1	9.0	9.2

ACS; Acute Coronary Syndrome, BMI; Body Mass Index, CCS; Chronic Coronary Syndrome, CKD; Chronic Kidney Disease, DM; Diabetes Mellitus, FFR; Fractional Flow Reserve, HTN; Hypertension, IVUS; Intravascular Ultrasound, NSTEMI; Non-ST elevation myocardial infarction; OCT; Optical Coherence Tomography, PAD; Peripheric Arterial Disease, STEMI; ST-elevation myocardial infarction, UA; Unstable Angina.

## Supplementary Materials

The following supporting information can be downloaded at: https://www.mdpi.com/article/10.3390/jcdd11020043/s1, Figure S1: PRISMA 2020 flow diagram for new-systematic reviews and meta-analyses, Figure S2: Evaluation of risk of bias of each RCT according to the Cochrane Collaboration Tool, Figure S3: Funnel Plots; Table S1: PICOS Approach, Table S2: Definitions for Each Endpoint, Table S3: Search strategy.
