# Supplementary material for: Efficacy and Safety of Thirty-Day Dual-Antiplatelet Therapy Following Complex Percutaneous Coronary Intervention: A Systematic Review and Meta-Analysis
Source: J Cardiovasc Dev Dis. 2024 Jan 29;11(2):43. doi: 10.3390/jcdd11020043 (PMC10889500; doi:10.3390/jcdd11020043)
Supplement: Supplementary file 1 [file jcdd-11-00043-s001.zip › jcdd-2792321-supplementary.pdf]

SUPPLEMENTARY MATERIAL

Supplementary Figure S1: PRISMA 2020 flow diagram for new-systematic reviews and meta-analyses

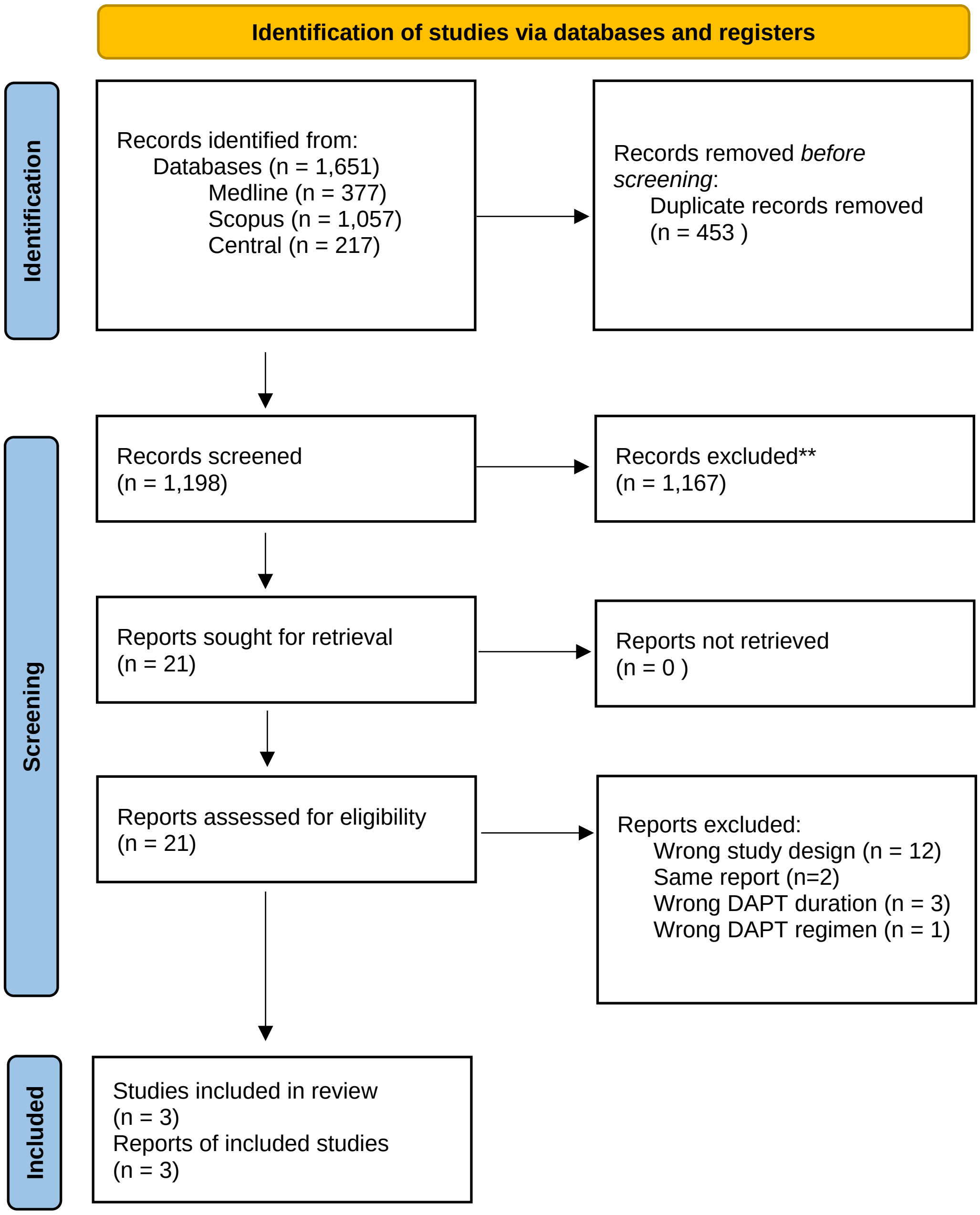

Supplementary Figure S2: Evaluation of risk of bias of each RCT according to the Cochrane Collaboration Tool.

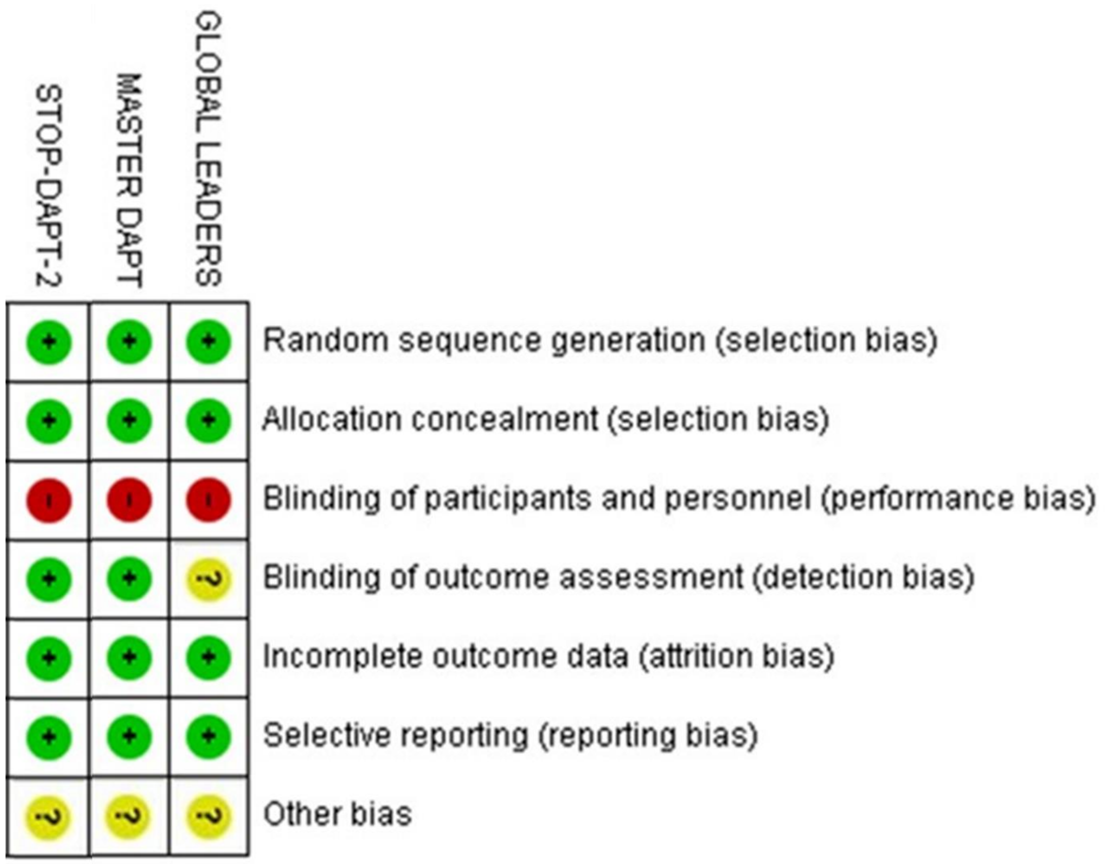

Supplementary Figure S3: Funnel Plots

Supplementary Figure S3A: Funnel Plot for Net Adverse Cardiovascular Events

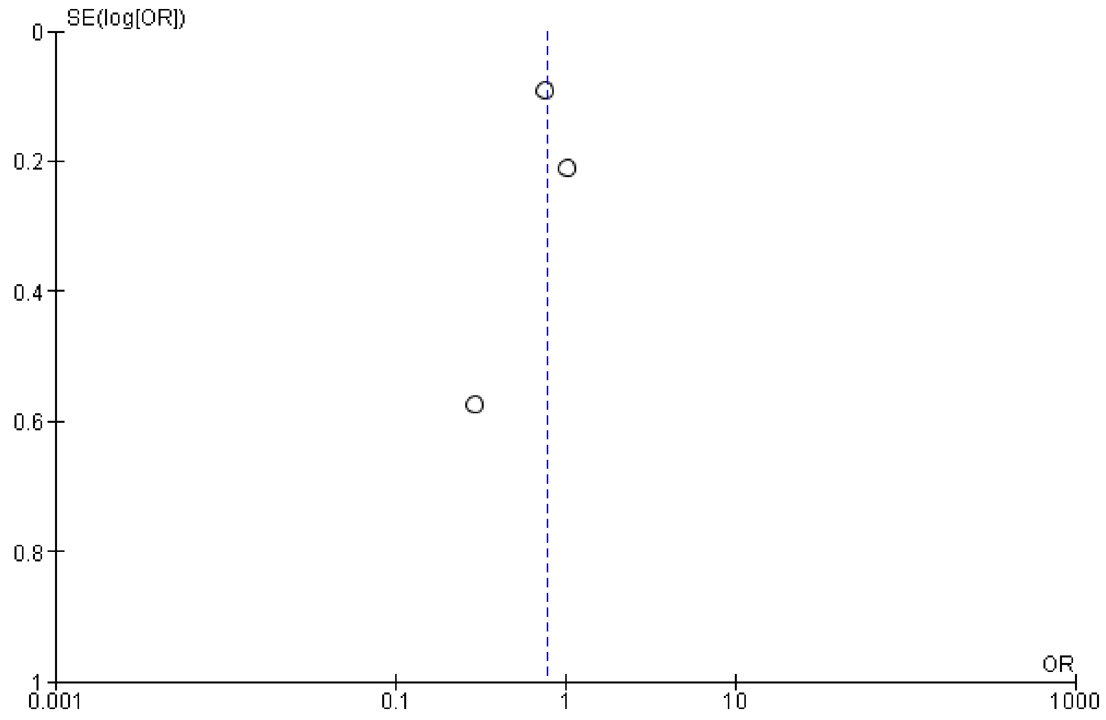

Supplementary Figure S3B: Funnel Plot for Major Adverse Cardiovascular Outcomes

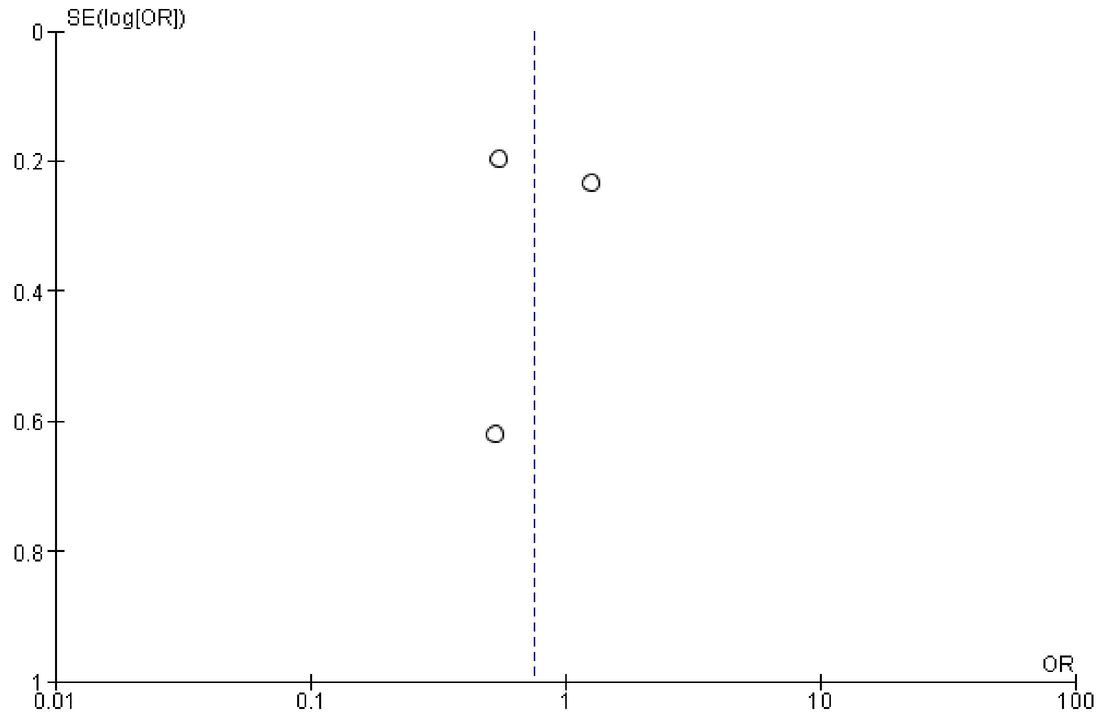

Supplementary Figure S3C: Funnel Plot for all – cause mortality

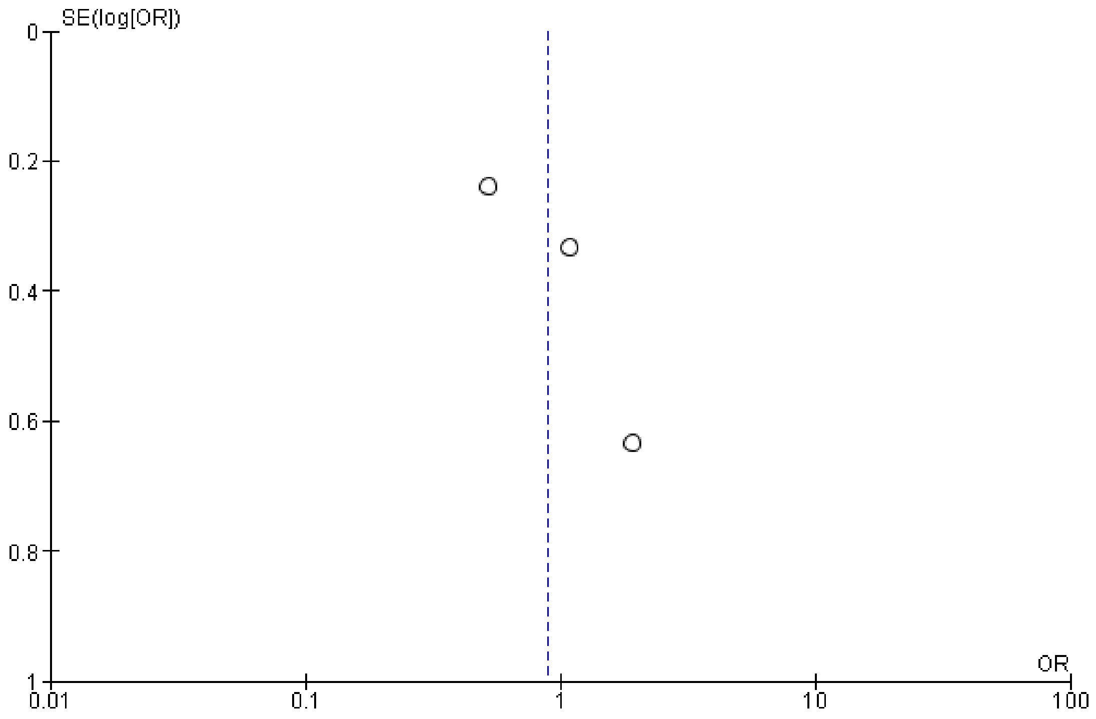

Supplementary Figure S3D: Funnel Plot for myocardial infarction

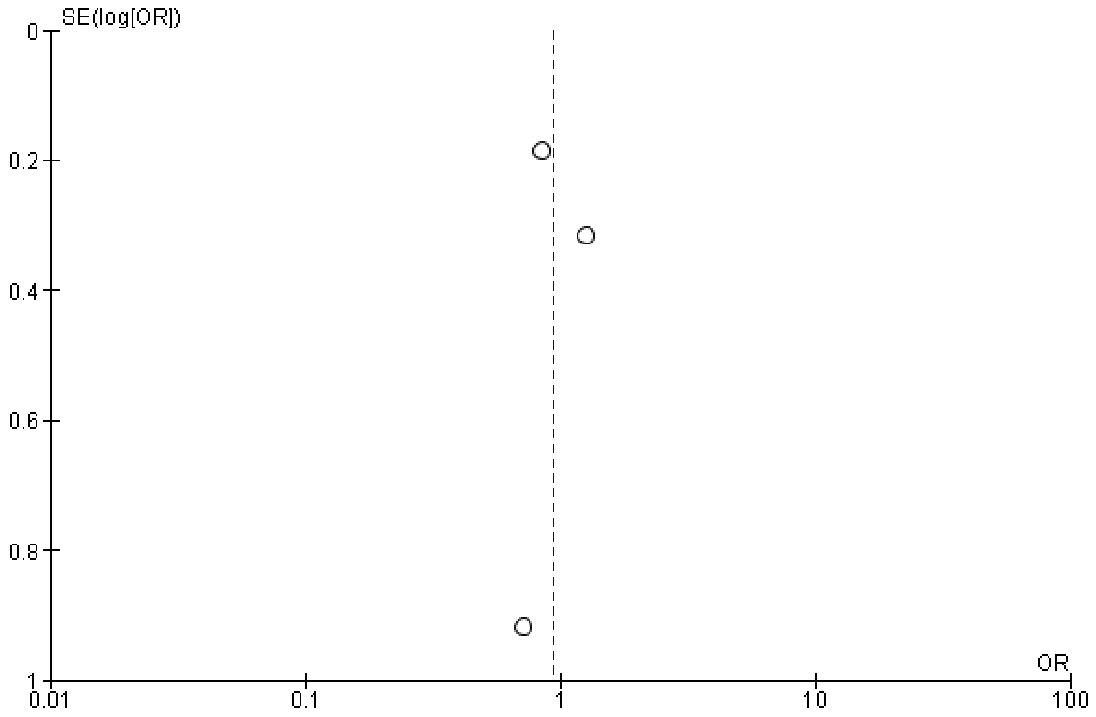

Supplementary Figure S3F: Funnel Plot for stroke

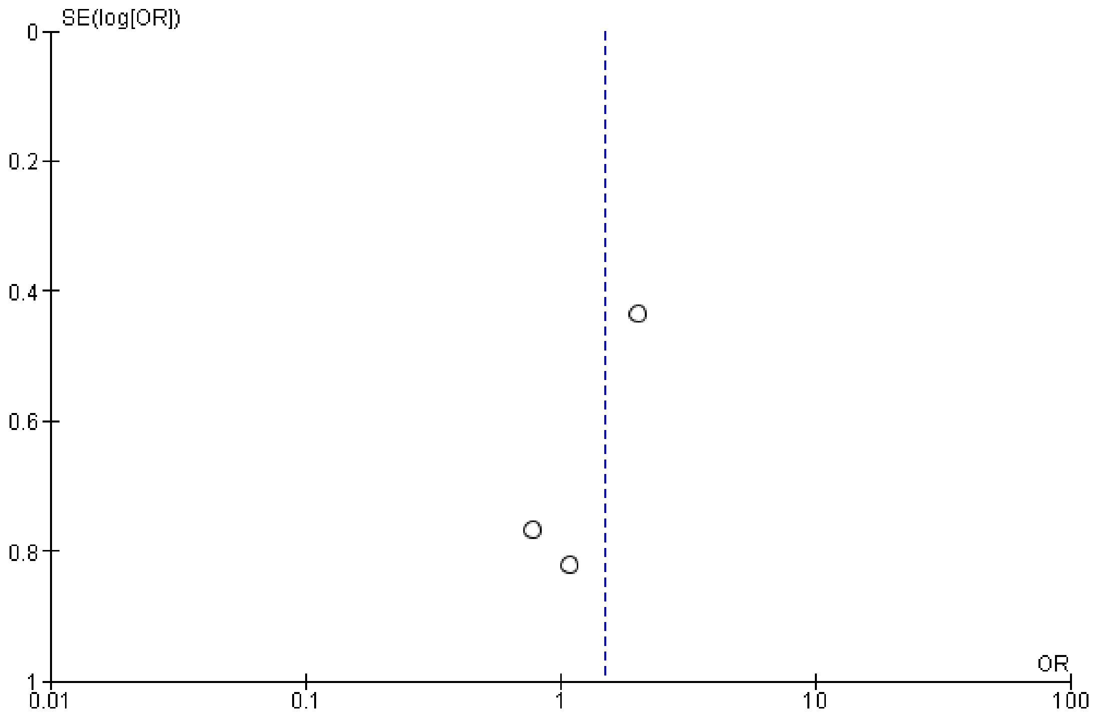

Supplementary Figure S3E: Funnel plot for stent thrombosis

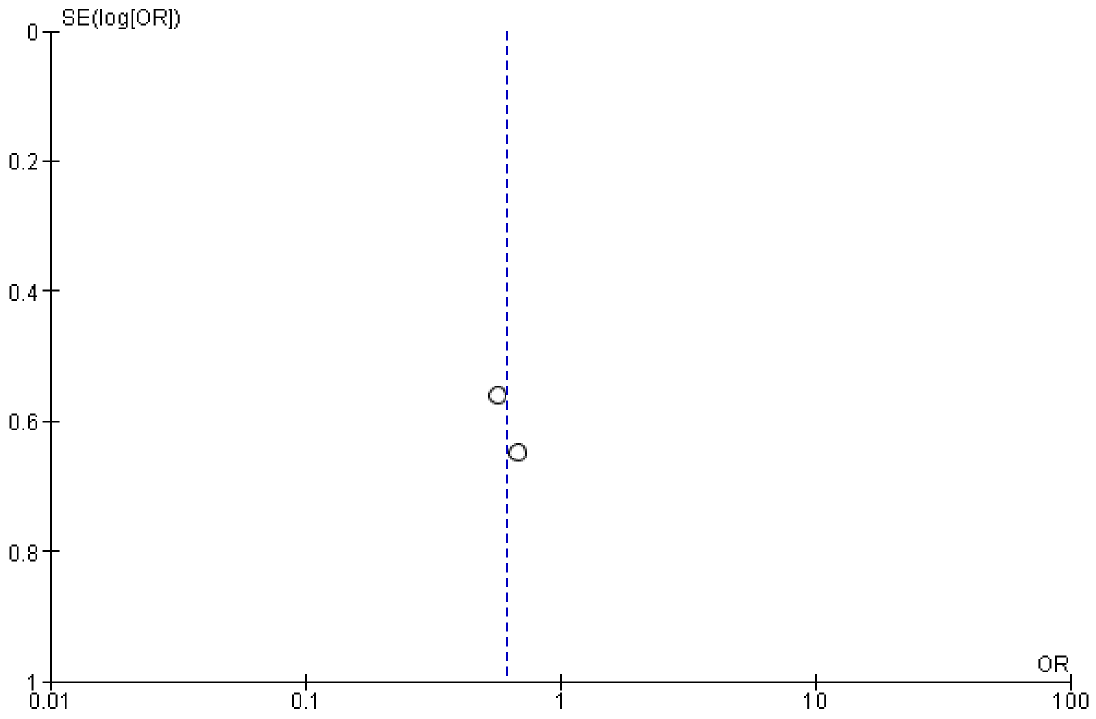

Supplementary Figure S3F Funnel plot for major bleedings

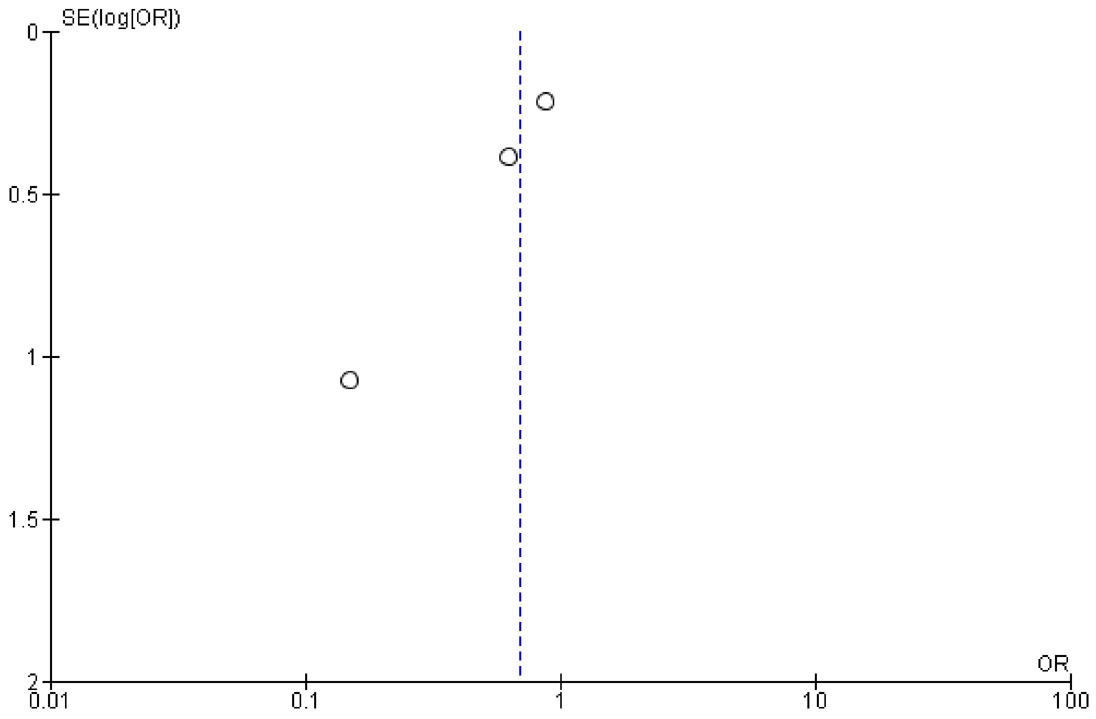
SE(log[OR])
0
0.5
1
1.5
2
OR
0.01
0.1
1
10
100

SUPPLEMENTARY TABLE S1: PICOS APPROACH	
**Population**	Adults (>18 years old) patients undergoing "complex PCI". The definition of complex PCI is not universal, so we used it as per trial defined and we reported them in Table 1.
**Intervention**	Dual antiplatelet therapy for 30 days, regardless P2Y12-inhibitor selection.
**Comparator**	Dual antiplatelet therapy for > 3 months, regardless P2Y12-inhibitor selection
**Outcome**	The primary endpoint of our study was to assess the incidence of net adverse clinical events (NACE), which is a composite outcome that includes both ischemic and bleeding events. Major Adverse Cardiovascular Events (MACE) is another composite outcome that includes death, non-fatal myocardial infarction, non-fatal stroke, and stent thrombosis; all the components of MACE were also used as secondary endpoints. Another secondary endpoint is major bleedings, classified as 3-5 according to Bleeding Academic Research Consortium (BARC) criteria. We preferred those over Thrombolysis in Myocardial Infarction (TIMI) or Safety and Efficacy of Enoxaparin in Percutaneous Coronary Intervention Patients, an International Randomized Evaluation (STEEPLE) criteria for improved consistency among the included reports. Taking into consideration the variability in outcome definitions across the

	included trials, a summary of these definitions is presented in Supplementary Table 2.
**Study Design**	Peer reviewed manuscripts or full conference proceedings reporting the primary results or subgroups including the population of interest of double blinded randomized controlled trials.

SUPPLEMENTARY TABLE S2: DEFINITIONS FOR EACH ENDPOINT					
**No**	**Trial**	**NACE**	**MACE**	**Major Bleeding**	**Stent Thrombosis**
1	**GLOBAL LEADERS**	all-cause mortality, any stroke, any MI any revascularization, or any bleeding BARC 3-5	All-cause mortality, stroke or new Q-wave MI	BARC 3-5	Definite
2	**STOPDAPT-2**	Cardiovascular death, MI, Definite ST, Stroke, or TIMI major or minor bleeding	Cardiovascular death, MI, definite stent thrombosis, or ischemic or hemorrhagic stroke	BARC 3-5	Definite or Probable
3	**MASTER-DAPT**	death from any cause, myocardial infarction, stroke, or major bleeding	All-cause mortality, MI or stroke	BARC 3-5	Definite or probable
BARC; Bleeding Academic Research Consortium, MACE; Major Cardiovascular Events, MI; Myocardial Infraction					

SUPPLEMENTARY TABLE S3: SEARCH STRATEGY		
**Database**	**Search Strategy**	**Results**
	#1 (dual antiplatelet):ti,ab,kw OR (dapt):ti,ab,kw	217

Cochrane Central Register of Controlled Trials (CENTRAL)	#2 ("percutaneous coronary intervention"):ti,ab,kw OR ("percutaneous coronary angioplasty"):ti,ab,kw OR ("percutaneous coronary revascularisation"):ti,ab,kw OR ("PCI"):ti,ab,kw OR (stent):ti,ab,kw	
	#3 (bifurcation):ti,ab,kw OR (left main):ti,ab,kw OR (multivessel):ti,ab,kw OR ("chronic total occlusion"):ti,ab,kw OR (complex):ti,ab,kw	
	#1 AND #2 AND #3	
SCOPUS	(TITLE-ABS-KEY ("dual antiplatelet" OR dapt) AND TITLE-ABS-KEY (bifurcation OR "LEFT MAIN" OR lm OR multivessel OR "CHRONIC TOTAL OCCLUSION" OR cto OR complex) AND TITLE-ABS-KEY ("percutaneous coronary intervention" OR pci OR stent))	1,057
MEDLINE	((("dual antiplatelet"[Title/Abstract]) OR (dapt[Title/Abstract])) AND (("percutaneous coronary intervention"[Title/Abstract]) OR (PCI[Title/Abstract]) OR ("stent"[Title/Abstract])) AND (("bifurcation"[Title/Abstract]) OR (left main[Title/Abstract]) OR ("multivessel"[Title/Abstract]) OR ("chronic total occlusion"[Title/Abstract]) OR ("cto"[Title/Abstract]) OR (“complex”[Title/Abstract]) OR (LM[Title/Abstract])))	377

SUPPLEMENTARY TABLE S4: INCLUSION, EXCLUSION CRITERIA AND ENDPOINTS OF EACH INCLUDED TRIAL					
	**Trial**	**Inclusion Criteria**	**Exclusion Criteria**	**Primary Endpoint**	**Secondary Endpoints**
1	GLOBAL LEADERS	Age ≥18 years, clinical indication for PCI, presence of one or more coronary artery stenosis of ≥50% in a native coronary artery or in a saphenous venous or arterial bypass conduit suitable for coronary stent implantation in a vessel with a reference vessel diameter of at least 2.25 mm	Intolerance to aspirin, P2Y12 inhibitors, bivalirudin, stainless steel or biolimus, known intake of a strong cytochrome P3A4 inhibitor as co-administration may lead to a substantial increase in exposure to ticagrelor, use of fibrinolytic therapy within 24 h of PCI, planned CABG as a staged procedure (hybrid) within 12 months of the index procedure, planned surgery within 12 months of PCI unless DAPT is	Composite of all-cause mortality or non-fatal centrally adjudicated MI	BARC (3 or 5), individual components of the primary endpoint; a composite endpoint of all-cause death, MI, or stroke, TVR or any revascularization;

			maintained throughout the peri-surgical period, need for oral anti-coagulation therapy, PCI for a priori known stent thrombosis, overt major bleeding, history of intracranial hemorrhage, stroke from ischemic or unknown cause within last 30 days		and definite stent thrombosis
2	STOPDAPT-2	Patients who have undergone PCI with the everolimuseluting cobalt-chromium stent (CoCr-EES, XienceTM) and have not experienced major complications (death, MI, stroke, or major bleeding) during hospital stay for treatment	DES other than Xience implanted in PCI performed at the time of enrollment, need for oral anticoagulation or antiplatelet therapy other than aspirin and P2Y12 inhibitors, history of intracranial bleeding, and known intolerance to clopidogrel	Composite of cardiovascular death, MI, ischemic or hemorrhagic stroke, definite stent thrombosis, or major or minor	Cardiovascular death, MI, ischemic or hemorrhagic stroke, or definite stent thrombosis, or major or minor bleeding

				bleeding at 12-month	
3	MASTER-DAPT	Age ≥18, at least one high bleeding risk criterion, all coronary lesions successfully treated with Ultimaster stent, free of any flow-limiting angiographic complications that required prolonged DAPT duration based on operator's decision, all stages of PCI were complete and no further PCI was planned, at least one high bleeding risk criterion (listed above) or on the basis of post-PCI actionable nonaccess-site	treated with stent other than Ultimaster stent within 6 months prior to index PCI, treated for in-stent restenosis or stent thrombosis at index PCI or within 6 months before, treated with a bioresorbable scaffold at any time prior to index procedure, incapable of providing written informed consent, under judicial protection, tutorship or curatorship, unable to understand and follow study-related instructions or unable to comply with study protocol, active bleeding requiring medical attention BARC ≥2 on randomization visit, life expectancy less than 1	a composite of death from any cause, myocardial infarction, stroke, or major bleeding, a composite of death from any cause, myocardial	A composite of death from cardiovascular causes, myocardial infarction, or stroke; all-cause death; stent thrombosis;

		related bleeding episode, uneventful 30-day clinical course (i.e. freedom from any new episode of ACS, symptomatic restenosis, stent thrombosis, stroke, any revascularization requiring prolonged DAPT) If not on OAC: a) Patient was on DAPT regimen of aspirin and a P2Y12 inhibitor; b) Patient with one type of P2Y12 inhibitor for at least 7 days 4. If on OAC: a) Patient was on the same type of OAC for at least 7 days; b)	year, known hypersensitivity or allergy to aspirin, clopidogrel, ticagrelor, prasugrel, cobalt chromium or sirolimus, any planned and anticipated PCI, participation in another trial, pregnant or breastfeeding women	infarction, or stroke	

		Patient was on clopidogrel for at least 7 days			

ACS; Acute Coronary Syndrome, BARC; Bleeding Academic Research Consortium, CABG; Coronary Artery Bypass Graft, CAD; Coronary Artery Disease, CKD; Chronic Kidney Disease, DAPT; Dual Antiplatelet treatment, DES; Drug Eluting Stent, DM; Diabetes Mellitus, LAD; Left Anterior Descending, MI; Myocardial Infraction, PCI; Percutaneous Coronary Intervention, TVR; Target Vessel Revascularization
